# Gut microbiota‐derived metabolite trimethylamine *N*‐oxide aggravates cognitive dysfunction induced by femoral fracture operation in mice

**DOI:** 10.1002/kjm2.12873

**Published:** 2024-07-04

**Authors:** Ying Xiong, Ya‐Nan Pu, Li‐Ya Li, Yang Su, Jia‐Yuan Niu, Zhao‐Yang Xiao

**Affiliations:** ^1^ Department of Anesthesiology The Second Affiliated Hospital of Dalian Medical University Dalian China

**Keywords:** inflammatory cytokine, postoperative cognitive dysfunction, quality of life, trimethylamine *N*‐oxide, underlying mechanisms

## Abstract

An increasing number of elderly individuals are experiencing postoperative cognitive dysfunction (POCD) problems after undergoing hip replacement surgery, with gut microbiota metabolites playing a role in its pathogenesis. Among these, the specific effects of trimethylamine *N*‐oxide (TMAO) on POCD are still unclear. This study aimed to explore the role of TMAO on cognitive dysfunction and underlying mechanisms in mice. The POCD model was created through femoral fracture surgery in elderly mice, followed by cognitive function assessments using the Morris Water Maze and Novel Object Recognition tests. The gut microbiota depletion and fecal microbiota transplantation were performed to examine the relationship between TMAO levels and cognitive outcomes. The effects of TMAO treatment on cognitive dysfunction, microglial activation, and inflammatory cytokine levels in the brain were also evaluated, with additional assessment of the role of microglial ablation in reducing TMAO‐induced cognitive impairment. Elevated TMAO levels were found to be associated with cognitive decline in mice following femoral fracture surgery, with gut microbiota depletion mitigating both TMAO elevation and cognitive dysfunction. In contrast, fecal microbiota transplantation from postoperative mice resulted in accelerated cognitive dysfunction and TMAO accumulation in germ‐free mice. Furthermore, TMAO treatment worsened cognitive deficits, neuroinflammation, and promoted microglial activation, which were reversed through the ablation of microglia. TMAO exacerbates cognitive dysfunction and neuroinflammation in POCD mice, with microglial activation playing a crucial role in this process. Our findings may provide new therapeutic strategies for managing TMAO‐related POCD and improving the quality of life for elderly patients.

## 1 INTRODUCTION

The incidence of hip fractures among the elderly is increasing due to the aging population. This is attributed to age‐related factors such as osteoporosis, reduced muscle strength, and decreased motor coordination.^1^ Despite effective hip replacement surgeries that may partially restore joint function, the most common complication of postoperative cognitive dysfunction (POCD) persists among these patients.^2^ POCD is characterized by reduced intellect, memory loss, impaired understanding, and psychological activity following surgery and anesthesia, lasting for weeks or months. In severe cases, patients may experience permanent cognitive impairments, leading to a loss of ability to perform daily activities independently.^3^ As a result, it is crucial to thoroughly explore the mechanisms of progression of POCD and to identify new therapeutic targets.

Various hypotheses have been proposed regarding the potential causes of POCD in the elderly, including the role of inflammatory responses associated with surgical stress and the potential neurotoxicity of anesthetic drugs.^4^, ^5^ Recently, an increasing body of evidence suggests that cognitive dysfunction may be linked to the gut microbiome, as the gut microbiota and its metabolites play an important role as regulators within the gut–brain axis.^6^ Specifically, the gut microbiota offers the host an array of metabolites that are initially absent, which are transported into the circulatory system and then modulate the microenvironment and functionality of the brain.^7^ For instance, intestinal flora dysbiosis can significantly impact the secretion of neurotransmitters within the brain, influencing host behavior and emotions,^8^ may promote the deposition of Aβ protein, resulting in cognitive dysfunction,^9^ and has the potential to modulate depression and anxiety disorders.^10^

Previous studies showed both alleviating and aggravating effects of gut microbiota metabolites on cognitive function. For example, butyrate has been demonstrated to support colonocyte energy metabolism and physiological homeostasis, potentially protecting against neurodegenerative processes.^11^ On the other hand, the level of propionic acid in human sera was linked to an elevated risk of cognitive decline. Similarly, recent studies have shown an association between plasma levels of t rimethylamine *N*‐oxide (TMAO) and cognitive dysfunction diseases.^12^ TMAO is produced in the liver through the oxidation of trimethylamine (TMA), a metabolite generated by the gut microbiota from dietary phosphatidylcholine and l‐carnitine. This oxidation process is facilitated by flavin‐containing monooxygenase 3 present in the liver.^13^ As a crucial metabolite derived from the gut microbiota, TMAO is implicated in neuroinflammation associated with aging and the decline in cognitive function mediated by astrocytes and microglia.^14^, ^15^ Furthermore, an elevation in TMAO levels in the circulation is linked to the downregulation of antioxidant enzymes in the hippocampus, which is associated with a decline in cognitive abilities.^16^ Furthermore, an increased breakdown of choline by *Escherichia coli*, whose abundance has been found in elderly mice exhibiting POCD, proved to be associated with an increase in the systemic levels of TMAO.^17^, ^18^ These findings offer indirect evidence of the association between TMAO and POCD. However, TMAO's direct effect on elderly mice undergoing femoral fracture surgery, their causal relationship, and the underlying intricate biological mechanisms remain unclear and necessitate further research.

In this research, an aged mouse model that underwent femoral fracture surgery was established to investigate the role of TMAO in the development of POCD. Moreover, we utilized strategies such as depletion and transplantation of gut bacteria in mice that received surgery and direct administration of TMAO to clarify the causal relationship between cognitive impairment with the TMAO levels. To investigate the regulatory mechanisms, TMAO was administered to POCD mice with pre‐interventions of microglial inactivation. By combining biochemical analyses with behavioral assessments, our study aims to offer a comprehensive understanding of the effect of TMAO on POCD progression in mice and investigate its underlying critical factor.

## 2 MATERIALS AND METHODS

### 2.1 Ethics statement

All experimental procedures were approved by the Animal Experiment Ethics Committee of Dalian Medical University (No: AEE19087). The housing environment was maintained with a 12 light/dark cycle ensuring that all animal experiments were designed with measures to reduce suffering. Euthanasia was performed humanely by administering an intraperitoneal injection of pentobarbital sodium at a dosage of 40 mg/kg at the end of the experiment.

### 2.2 Animal experimental schedule

A total of 136 elderly C57BL/6 mice (18–24 months, 25–30 g) and germ‐free C57BL/6 mice (18–24 months, 25–30 g) utilized for modeling POCD and fecal microbiota transplantation (FMT), respectively, were purchased from the Laboratory Animal Center of China Medical University (Shenyang, China). In this study, a sequential experimental approach was developed to investigate the impact of TMAO on cognitive dysfunction post‐femoral fracture in mice. The study started with cognitive function assays and TMAO level measurements performed on elderly mice following femoral fracture surgery. Following this, a subset of mice received a week of antibiotic (ABX) pretreatment before surgery. Furthermore, fecal microbiota from standard or surgery models was transplanted into germ‐free mice after 4 h of surgery to explore the gut microbiota's role in neuroinflammation and cognitive impairment. Another group of mice underwent TMAO treatment for 3 weeks before femoral fracture surgery to evaluate the compound's direct effect. To assess the essential role of microglia in TMAO‐exacerbated cognitive deficits, elderly mice were pretreated with PLX5622 before TMAO administration for microglia inactivation.

The ABX regimen consisted of ampicillin (1 g/L), vancomycin (0.5 g/L), neomycin sulfate (1 g/L), and metronidazole (1 g/L). These reagents were dissolved in the drinking water of the mice to ensure continuous exposure throughout the pretreatment period. The TMAO was administrated orally at a dosage of 180 mg/kg every day during the intervention process. For microglia inactivation, PLX5622 (Plexxikon Inc., Berkeley, CA, USA) was delivered at a dosage of 1200 ppm incorporated into the rodent chow. The mice had ad libitum access to the PLX5622‐enriched feed before the initiation of surgical procedures.

Following these pretreatments, mice received femoral fracture surgery, following which cognitive function, TMAO levels, and neuroinflammatory parameters were evaluated.

### 2.3 Femoral fracture operation

Following the induction of anesthesia, the surgical site was disinfected with 70% ethanol and povidone‐iodine. A longitudinal incision was then made over the lateral aspect of the femur, and a stainless‐steel intramedullary pin (diameter: 0.25 mm; Synthes, Paoli, PA, USA) was introduced into the femoral canal to stabilize the fracture. The femoral fracture was created using a precision bone cutter (Stryker, Kalamazoo, MI, USA). Following the fracture, the muscle layers and skin were sutured and mice were administered through subcutaneous injections of buprenorphine every 12 h for 48 h to manage pain.^19^ In the Sham control, mice underwent a surgical procedure that mimicked the steps of the experimental surgery without actually conducting a femoral fracture. The incidence rate of POCD in the animal model following the femoral fracture operation was 50% and only those showing cognitive dysfunction were included in the POCD group.

### 2.4 Morris water maze (MWM) test

A circular pool (diameter: 120 cm; depth: 50 cm; Stoelting Co., Wood Dale, IL, USA) was filled with water maintained at a temperature of 22 ± 1°C. A nontoxic, water‐soluble dye (Brilliant Blue FCF; Sigma‐Aldrich, Shanghai, China) was dissolved in the water to ensure optimal opacity. The pool was divided into four equal quadrants, and a platform (diameter: 10 cm) was placed in one quadrant, submerged 1 cm below the water surface. Training sessions were performed over five consecutive days, during which mice were gently introduced into the pool from randomized starting positions along the perimeter. The time taken by the mice to find the hidden platform was noted as the escape latency. After training, the platform was removed during the probe trials following training to evaluate the retention of spatial memory, during which the number of platform crossings and the time spent in the target quadrant were recorded. The movements of mice were tracked utilizing a high‐definition video tracking system (Stoelting Co., Wood Dale, IL, USA) positioned above the center of the pool.

### 2.5 Novel object recognition (NOR) test

After the femoral fracture surgery intervention, mice were individually placed into an experimental arena measuring 60 × 40 × 40 cm for the NOR test.^20^ During the initial exposure phase, subjects were given a 10‐min exploration period of two identical objects strategically positioned at diametric ends of the enclosure. After 24 h, the experimental paradigm was modified by replacing one of the previously presented objects with a new item. Behavioral responses during these sessions were recorded utilizing a high‐definition video recording apparatus (Sony HDR‐CX405, Tokyo, Japan) to quantify the duration assigned to each object. The exploration index was calculated by: time spent exploring the novel object/total exploration time.

### 2.6 High‐performance liquid chromatography‐mass spectrometry (HPLC‐MS)

The quantification of TMAO concentrations in murine plasma, brain, and intestinal tissues was conducted using an HPLC‐MS approach.^21^ Obtained samples were prepared through homogenization in phosphate‐buffered saline (PBS), followed by centrifugation at 10,000 *g* for 10 min at 4°C to obtain supernatants. The supernatants were subsequently deprotenized using methanol (Sigma‐Aldrich) at a ratio of 1:4, followed by a second centrifugation under the same conditions. The analysis was performed using an Agilent 1290 Infinity II HPLC system coupled with an Agilent 6495 Triple Quadrupole MS detector (Agilent Technologies, Santa Clara, CA, USA), employing a Zorbax Eclipse Plus C18 column (2.1 mm × 100 mm, 1.8 μm particle size; Agilent Technologies) for the separation. The mobile phase comprised a mixture of 0.1% formic acid in water (Solvent A) and acetonitrile (Solvent B, HPLC grade; Sigma‐Aldrich) with a mass spectrometer operated in positive ion mode. TMAO standard was purchased from Sigma‐Aldrich, and testing procedures were conducted based on the standard protocols of instruction manuals provided by the reagent manufacturer.

### 2.7 Enzyme‐linked immunosorbent assay (ELISA)

The cytokines IL‐1β (P1301), IL‐6 (P3264), TNF‐α (PT512), and IL‐10 (PI522) were measured using specific ELISA kits purchased from the Beyotime Institute of Biotechnology (Jiangsu, China). The brain tissues of mice were homogenized in a lysis buffer, and the protein concentrations were determined using a BCA Protein Assay Kit (Pierce Biotechnology, Rockford, IL, USA). The homogenates were then diluted and evaluated as per the manufacturer's instructions. The absorbance was measured at a wavelength of 450 nm using a microplate reader (Bio‐Rad Laboratories, Hercules, CA, USA).

### 2.8 Hematoxylin and eosin (H&E) staining

To conduct the pathological examination of the liver, the formalin‐fixed, paraffin‐embedded sections were subjected to H&E staining, and the pathological changes were observed under a light microscope.

### 2.9 Immunohistochemistry (IHC)

IHC for the microglial marker Iba‐1 was conducted on murine brain tissue sections to characterize microglial activation states.^22^ The brain tissues were initially fixed in 4% paraformaldehyde (Sigma‐Aldrich) and then embedded in paraffin. Sections with a thickness of 5 μm were cut and mounted on silane‐coated slides. The tissue sections were deparaffinized in xylene and rehydrated through a graded ethanol series. Antigen retrieval was obtained by heating the sections in sodium citrate buffer (10 mM, pH 6.0; Sigma‐Aldrich) at 95°C for 20 min. After quenching endogenous peroxidase activity with 3% hydrogen peroxide (Sigma‐Aldrich) in methanol for 15 min, the sections were blocked at room temperature for 1 h. Subsequently, the sections were incubated with a primary antibody against Iba‐1 (1:500 dilution; Sigma‐Aldrich) overnight at 4°C in a humidified chamber, followed by incubation with a biotinylated secondary antibody (Sigma‐Aldrich) for 1 h at room temperature. Stained sections were counterstained with hematoxylin, dehydrated, and mounted with a xylene‐based mounting medium (Sigma‐Aldrich), followed by examined under a light microscope (BX53; Olympus Corporation, Tokyo, Japan).

### 2.10 Flow cytometry

Isolated microglial cells were resuspended in PBS and blocked with Fc Block (BD Biosciences, San Jose, CA, USA). Subsequently, they were incubated with fluorescein isothiocyanate (FITC)‐conjugated anti‐CD86 antibody (Clone GL‐1; BioLegend, San Diego, CA, USA) and allophycocyanin‐conjugated anti‐CD206 antibody (Clone C068C2; BioLegend) for 30 min at 4°C in the dark. The antibodies were employed at a concentration of 1: 500. Following incubation, cells were washed with PBS containing 2% fetal bovine serum to remove unbound antibodies. Subsequently, the samples were obtained on a flow cytometer (BD Biosciences), and data were analyzed using FlowJo software (FlowJo LLC, Ashland, OR, USA).

### 2.11 Statistical analysis

The graphical representations and statistical computations were performed by GraphPad Prism (Version 8.0; GraphPad Software, San Diego, CA, USA). The distribution of data was initially assessed for normality using the Shapiro–Wilk test. Depending which one‐way analysis of variance with subsequent post hoc analysis via Tukey's test or the Kruskal–Wallis test, followed by Dunn's multiple comparisons test was employed. Correlational analyses to investigate the relationship between escape latency, the number of platform crossings, the exploration index, and the concentrations of TMAO in the gut were performed using Pearson's correlation coefficient for parametric data or Spearman's rank correlation coefficient for non‐parametric data. Quantitative variables were reported as mean ± standard error of the mean (SEM). Statistical significance was set at a *p*‐value less than 0.05.

## 3 RESULTS

### 3.1 Elevated TMAO levels correlate with cognitive decline in mice post‐femoral fracture operation

**FIGURE 1:**
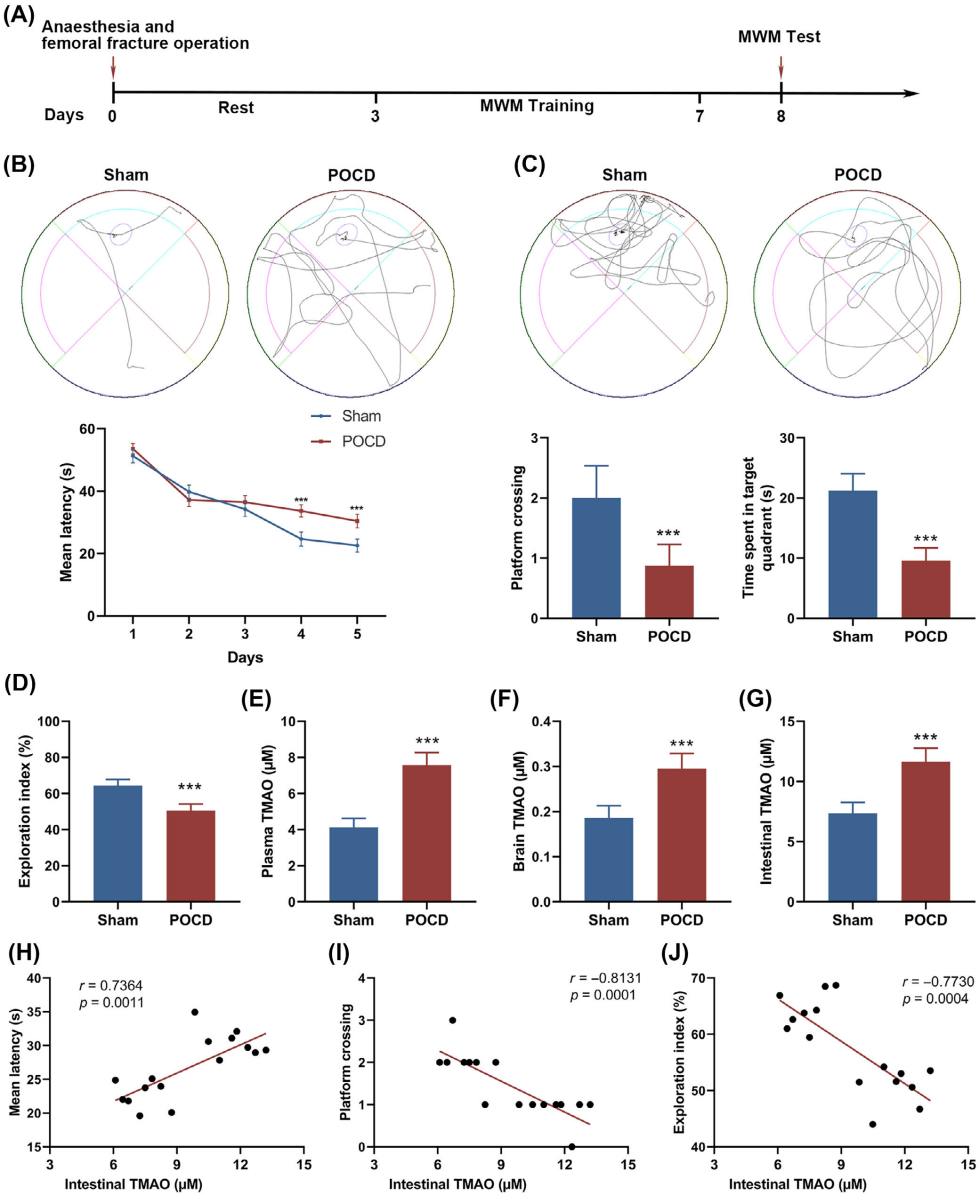
Elevated cerebral TMAO levels was observed and associated with cognitive dysfunction in femoral fracture operation mouse model. (A) A schematic representation of the experimental timeline, showing the establishment of the femoral fracture model, followed by water maze navigation training from the 3rd to the 7th‐day post‐operation, and concluding with a space exploration test on the 8th day. (B) The mean latency, (C) number of platform crossings, and time spent in the target quadrant of mice in the Morris water maze test. (D) The novel object recognition was subsequently performed and the exploration index of new objects was calculated to determine recognition memory deficits. (E) The levels of TMAO in the (E) plasma, (F) brain, and (G) intestines of POCD mice were assessed through high‐performance liquid chromatography‐mass spectrometry and the correlation between intestinal TMAO concentrations with (H) escape latency, (I) the number of crossings over the target zone and (J) the exploration index for novel objects were examined by the Pearson's correlation test. Data was represented as mean ± SEM. ***p < 0.001.

We established a POCD model through femoral fracture surgery and analyzed the relationship between TMAO levels and cognitive dysfunction in mice. Commencing the 3rd day after establishing the fracture model, water maze navigation training was carried out for five consecutive days, followed by the space exploration test on the eighth day (Figure 1A). The MWM test showed significant cognitive impairments in the mice with femoral fracture surgery, demonstrated by an elevated mean latency to locate a hidden platform during the navigation trials starting from day 4 post‐operation (Figure 1B). Particularly, upon removing the escape platform on day 8, mice from the femoral fracture group displayed both reduced numbers of crossings over the former platform location and shorter dwell times, indicating diminished spatial memory retention (Figure 1C). Furthermore, the NOR test showed a significant decrease in sensitivity to new objects among the femoral fracture mice compared to the Sham control, indicating impairments in recognition memory (Figure 1D).

In mice of the POCD group, an increase in TMAO levels was found in their plasma (Figure 1E), brain (Figure 1F), and intestines (Figure 1G) in comparison to the Sham group. Furthermore, correlation analyses showed that intestinal TMAO concentrations were positively associated with escape latency (Figure 1H) and inversely related to the number of crossings over the target zone (Figure 1I) and the exploration index of novel objects (Figure 1J). These findings collectively indicate that increased TMAO levels are associated with the manifestation of POCD in mice.

### 3.2 Gut microbiota depletion mitigates TMAO elevation and cognitive dysfunction in mice post‐femoral fracture operation

**FIGURE 2:**
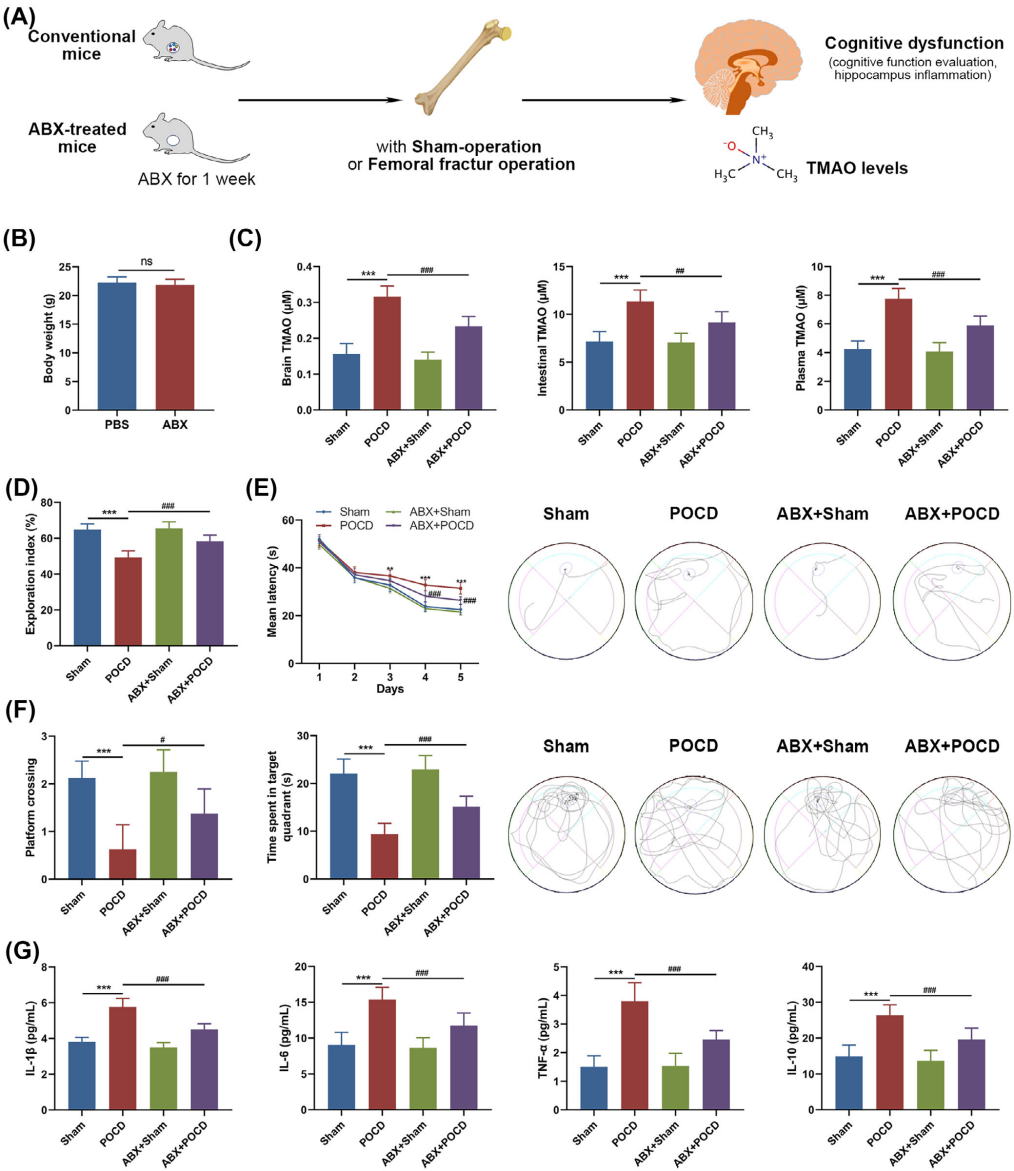
Impact of gut microbiota depletion on TMAO levels and cognitive dysfunction in a femoral fracture operation mouse model. (A) Schematic representation of the experimental setup, showing a week‐long antibiotic (ABX) pretreatment before femoral fracture surgery to evaluate effects on TMAO concentrations, cognitive performance, and neuroinflammation. (B) Comparison of body weights between ABX‐treated and PBS‐treated control mice. (C) TMAO concentrations in brain, intestines, and plasma following femoral fracture operation. (D) Novel object recognition test and (E, F) Morris water maze test were performed to assess cognitive function affected by gut microbiota depletion. (G) ELISA analysis was employed to detect inflammatory markers in brain tissues. Data represented as mean ± SEM, with **p < 0.01, ***p < 0.001 between the Sham and POCD group; ^#^ *p* < 0.05, ^##^ *p* < 0.01, ^###^ *p* < 0.001 between the POCD and ABX + POCD group; ns, no significance.

To evaluate the influence of gut microbiota, mice were administered antibiotics prior to femoral fracture surgery. The schematic of the experiment (Figure 2A) entailed a week of ABX pretreatment in mice, followed by an evaluation of the effect of femoral fracture surgery on TMAO concentrations, cognitive function, and hippocampus inflammation levels. The ABX treatment did not significantly change mouse body weight when compared to the PBS‐treated control (Figure 2B). However, the concentration of TMAO in the brain, intestine, and plasma (Figure 2C) was notably reduced in the POCD mice after ABX pretreatment, indicating a link between gut microbiota and TMAO levels post‐surgery. The NOR test showed that the exploration index of the POCD group was lower than the Sham, which was reversed in the ABX + POCD group (Figure 2D). During the MWM trial, from the fourth day of training, the ABX + POCD group exhibited a significantly lower escape latency compared to the POCD group (Figure 2E), indicating improved cognitive performance. Moreover, the number of crossings over the original platform location and the time spent there were significantly higher in the ABX‐treated POCD mice compared to the non‐ABX‐treated counterparts (Figure 2F). ELISA analysis of brain tissue inflammatory markers showed elevated levels in POCD mice compared to the Sham (Figure 2G). Intriguingly, depletion of gut microbiota effectively reduced the surge in these inflammatory factors post‐femoral fracture surgery, with levels of IL‐1β, IL‐6, TNF‐α, and IL‐10 all being lower in the ABX pretreated group (Figure 2G).

### 3.3 Fecal microbiota transplantation from femoral fracture operation mice accelerates cognitive dysfunction and TMAO accumulation in germ‐free mice

**FIGURE 3:**
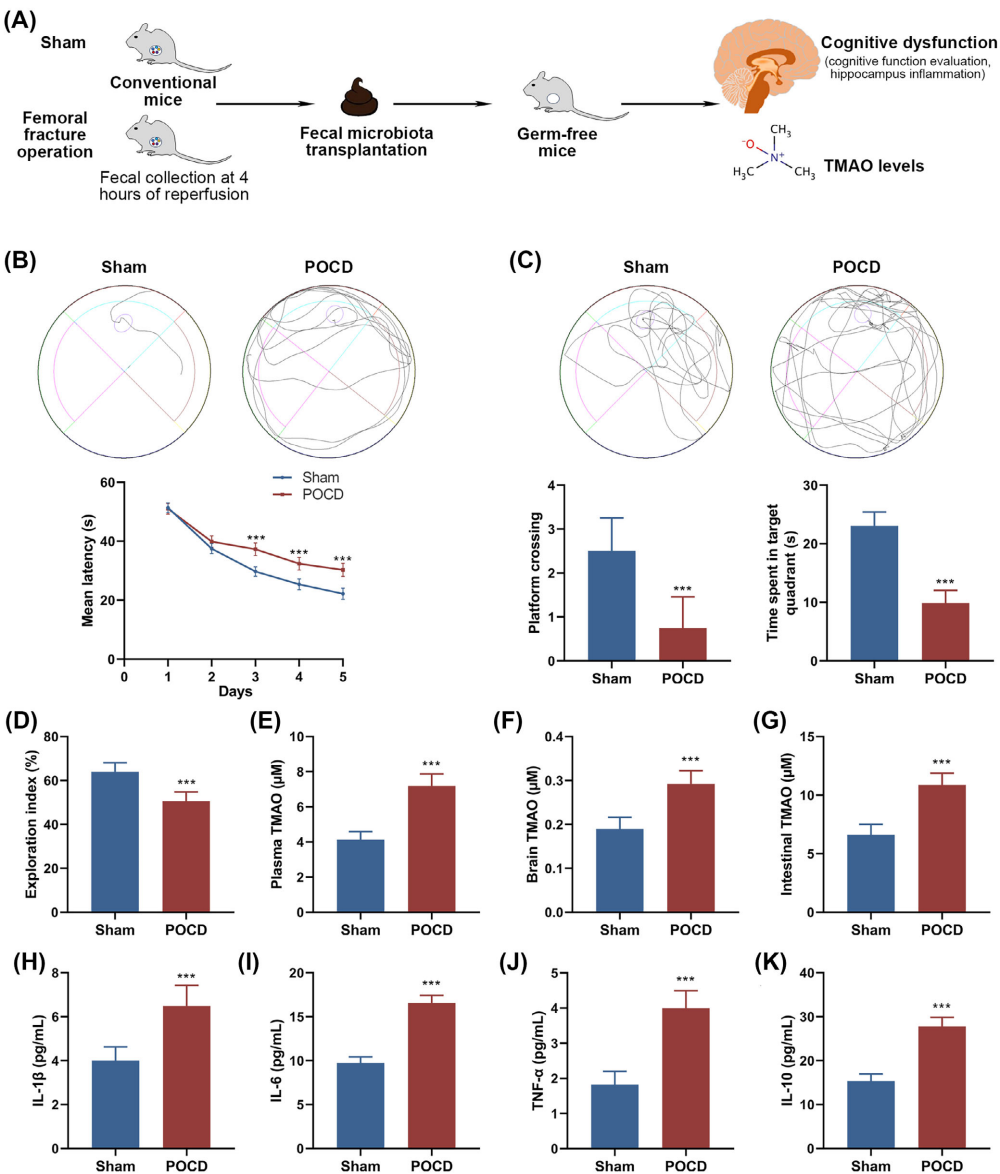
Acceleration of cognitive dysfunction and TMAO accumulation in germ‐free mice through fecal microbiota transplantation (FMT) from femoral fracture‐operated mice. (A) A schematic illustration of the FMT process from donors (normal or femoral fracture operation‐treated mice) to germ‐free recipients. Following FMT, the cognitive function of mice was evaluated through (B) escape latency, (C) the number of platform crossings, time spent in the target quadrant, and (D) the exploration index utilizing the Morris water maze test or novel object recognition test. The TMAO levels in (E) plasma, (F) brain, and (G) intestines were identified to determine the effect of FMT on TMAO accumulation. (H–K) Proinflammatory cytokines demonstrate the inflammatory profiling of brain tissues after FMT from femoral fracture operation‐treated mice. Data are presented as mean ± SEM. Statistical significance is denoted by ***p < 0.001.

Microbial transplants were obtained from the feces of normal or femoral fracture operation‐treated mice 4 h post‐surgery (Figure 3A). The cognitive functions of the recipient mice, along with TMAO concentrations and inflammatory markers in the brain, were subsequently evaluated. In the MWM, starting from day 3 of training, mice undergoing the postoperative microbiota (POCD group) showed significantly longer escape latency periods compared to the Sham group, indicating impaired spatial learning (Figure 3B). Furthermore, when the escape platform was removed, POCD mice exhibited a notable decrease in the number of platform crossings and time spent in the target quadrant, indicating memory retention deficits (Figure 3C). The NOR test further validated cognitive decline, as shown by a reduced exploration index in POCD mice compared to the Sham (Figure 3D). Analysis of TMAO levels in plasma (Figure 3E), brain (Figure 3F), and intestines (Figure 3G) showed significantly higher concentrations in the POCD group than in the control. Moreover, inflammatory profiling showed increased levels of pro‐inflammatory cytokines in the brain tissues of POCD mice (Figure 3H–K). These findings indicate that FMT from mice treated with femoral fracture operations significantly contributes to cognitive dysfunction and systemic TMAO accumulation in germ‐free mice.

### 3.4 TMAO treatment exacerbates cognitive dysfunction and promotes microglial activation in femoral fracture operation mice

**FIGURE 4:**
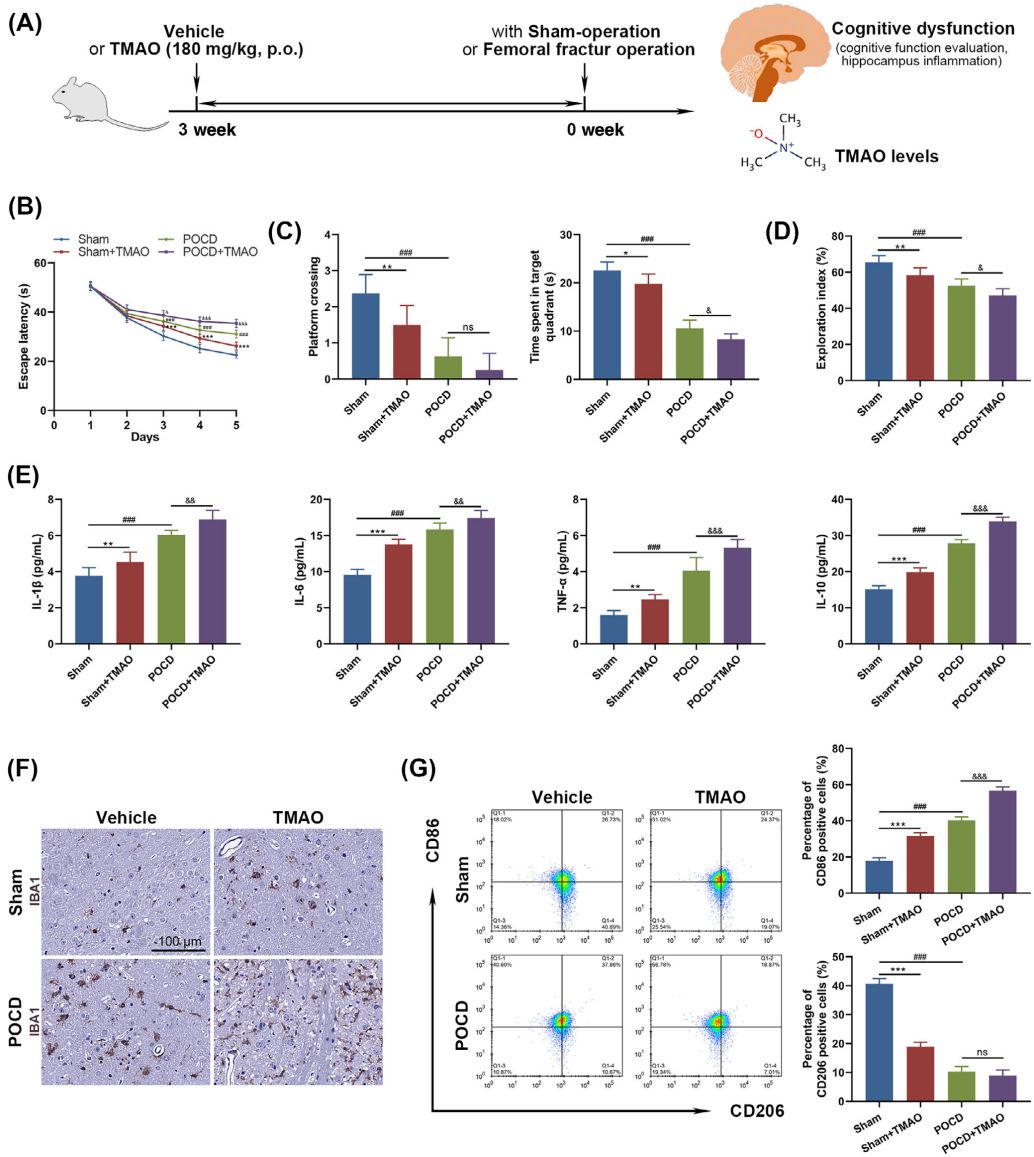
Effects of TMAO treatment on cognitive dysfunction and microglial activation in a femoral fracture operation mouse model. (A) Experimental design showing oral administration of TMAO at a dose of 180 mg/kg for 3 weeks before femoral fracture surgery. (B, C) Morris water maze test and (D) the novel object recognition test revealed diminished exploration indexes in TMAO‐treated groups. (E) Increased levels of inflammatory cytokines IL‐1β, IL‐6, TNF‐α, and IL‐10 in brain tissues indicate increased neuroinflammation due to TMAO exposure. (F) Immunohistochemistry for Iba‐1 was carried out to detect microglial activation, characterized by denser brown precipitates. (G) Flow cytometry analysis on CD86 and CD206 positive cells was conferred to reveal microglial polarization. Data are demonstrated as mean ± SEM. *p < 0.05, **p < 0.01, ***p < 0.001 (Sham vs. Sham + TMAO); ^###^ *p* < 0.001 (Sham vs. POCD); ^&^ *p* < 0.05, ^&&^ *p* < 0.01, ^&&&^ *p* < 0.001 (POCD vs. POCD + TMAO); ns, no significance.

To elucidate the TMAO's effects on POCD mice, we administered that at a dose of 180 mg/kg orally for 3 weeks before femoral fracture surgery in mice (Figure 4A). Given the potential for oral administration of TMAO to induce liver injury, we examined the liver. The H&E staining revealed no necrosis or liver injury in the liver sections of all groups' mice (Figure S1A). Starting from day 3 of training of the MWM test, noticeable differences in escape latency were detected among the groups, with the TMAO‐treated mice revealing the longest latency, followed by POCD, Sham + TMAO, and Sham (Figure 4B). Moreover, POCD mice spent more time in the target quadrant compared to POCD + TMAO mice (Figure 4C). Similar patterns were noted in the exploration index (Figure 4D), further indicating the worsened cognitive impairments induced by TMAO. Inflammatory cytokine levels in brain tissues, such as IL‐1β, IL‐6, TNF‐α, and IL‐10, were notably heightened in TMAO‐treated groups compared to their respective controls, signifying an enhanced inflammatory response due to TMAO exposure (Figure 4E). Furthermore, TMAO levels in plasma (Figure S1B), brain (Figure S1C), and intestines (Figure S1D) of the Sham + TMAO group exhibited significantly higher concentrations compared to the control, resembling those of the POCD. Administration of TMAO further increased the TMAO levels in plasma (Figure S1B), brain (Figure S1C), and intestines (Figure S1D) of POCD mice.

IHC for Iba‐1 showed elevated microglial activation in TMAO‐treated groups, with denser brown precipitates found in POCD + TMAO than POCD, suggesting a TMAO‐mediated increase in microglial activation (Figure 4F). Flow cytometry analysis of microglial polarization markers showed a similar trend, with a higher presence of CD86‐positive cells in the TMAO‐treated groups, while CD206‐positive cells were less abundant in Sham + TMAO compared to Sham. These show an imbalance in microglial polarization toward a more inflammatory phenotype (Figure 4G). These findings indicate that TMAO might elevate neuroinflammation and change microglial polarization states through which exacerbating cognitive dysfunction in POCD mice.

### 3.5 Microglial activation is crucial in TMAO‐induced cognitive dysfunction during femoral fracture operations

**FIGURE 5:**
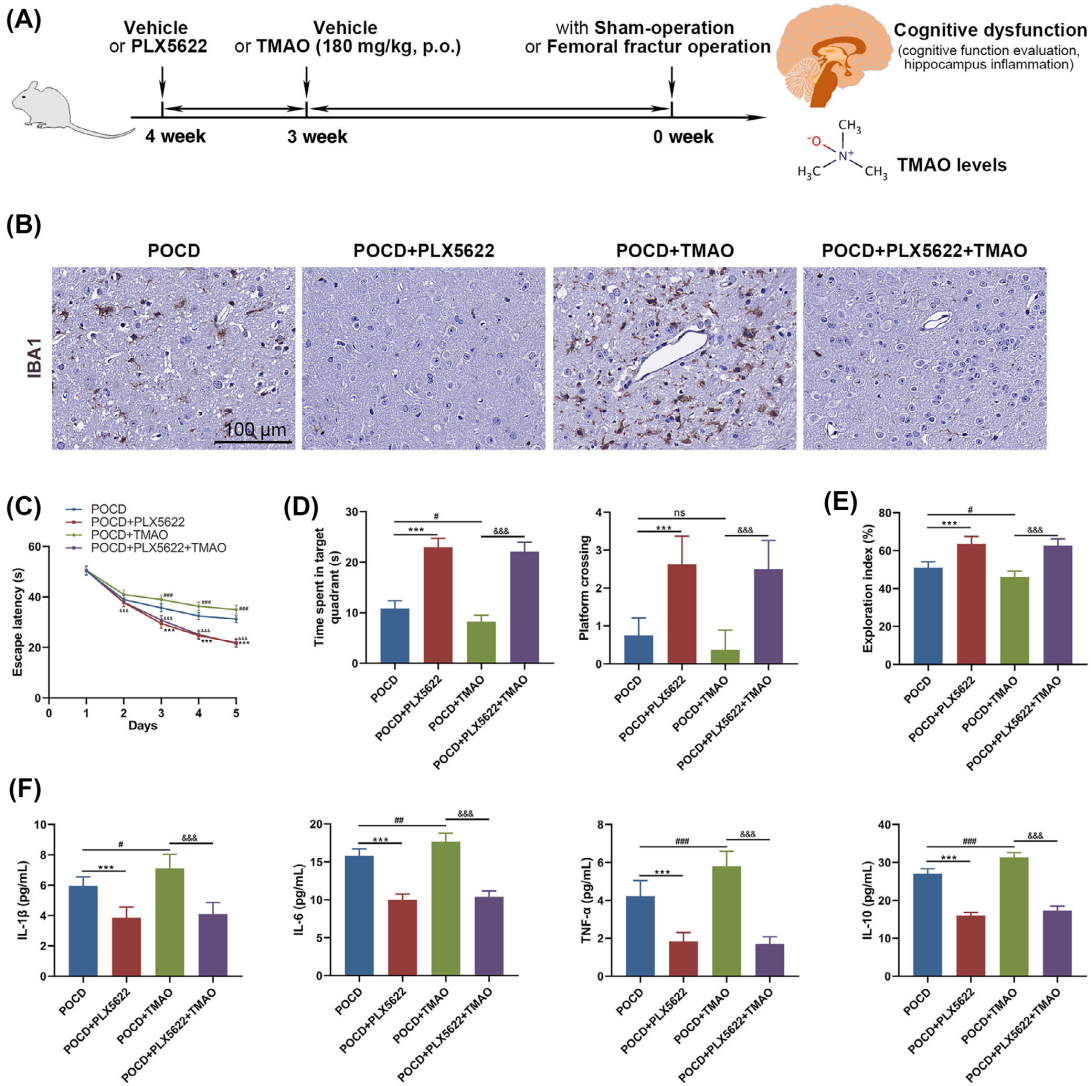
Microglia activation was essential for TMAO‐mediated cognitive dysfunction during femoral fracture operation. (A) Diagram illustrating PLX5622 administration for one week before TMAO treatment to inactivate microglia. (B) Immunohistochemistry for Iba‐1 in brain sections. (C) Escape latency, (D) frequency of platform crossings and duration spent in the target platform location were obtained from the Morris water maze test. (E) NOR test was utilized to show exploration indexes of mice for new objects. (F) The inflammatory cytokine levels in brain tissues were detected to determine neuroinflammation via ELISA. Data was represented as mean ± SEM. ***p < 0.001 (POCD vs. POCD + PLX5622); ^#^ *p* < 0.05, ^##^ *p* < 0.01, ^###^ *p* < 0.001 (POCD vs. POCD + TMAO); ^&&&^ *p* < 0.001 (POCD + TMAO vs. POCD + PLX5622 + TMAO); ns, no significance.

To investigate the impact of microglial activation in exacerbating cognitive impairment caused by TMAO, mice were pretreated with PLX5622 for one week prior to TMAO administration to deactivate microglia (Figure 5A). IHC for Iba‐1 in brain sections revealed that PLX5622 pretreatment successfully reversed microglial activation exacerbated by TMAO (Figure 5B).

Cognitive function evaluation showed significant disparities from the third day of training, where the escape latency was significantly decreased in the POCD + PLX5622 + TMAO mice compared to the TMAO‐treated POCD mice without PLX5622 pretreatment (Figure 5C). Moreover, the frequency of platform crossings, the duration of time spent in the target platform location (Figure 5D), and the exploration index for new objects (Figure 5E) were significantly higher in the POCD + PLX5622 + TMAO group than in the POCD + TMAO group, indicating a considerable alleviation of cognitive deficits. The levels of inflammatory cytokines in brain tissues indicated that PLX5622 pretreatment substantially reversed the heightened levels found in the POCD + TMAO group compared to the POCD group (Figure 5F), showcasing a notable reduction in neuroinflammation when microglia were inactivated. These findings emphasize the crucial role of microglial activation in the mechanism by which TMAO exacerbates cognitive dysfunction in a femoral fracture surgery model.

## 4 DISCUSSION

POCD is a common complication affecting a significant proportion of patients, particularly the elderly, following surgery, resulting in reduced quality of life and prolonged recovery times.^4^, ^23^ The elucidation of the role of the gut metabolome in changing cognitive dysfunction post‐surgery offers valuable insights into the pathophysiology of POCD and presents potential strategies for therapeutic interventions. Our study elucidates the complex relationship between TMAO and cognitive dysfunction following femoral fracture surgery in murine models. It underscores the importance of TMAO restriction in improving cognitive deficits by deactivating microglia and reducing neuroinflammation.

Dietary intake of choline and carnitine, which are abundantly present in red meat, eggs, dairy products, and fish, undergoes microbial breakdown in the gastrointestinal tract, leading to the formation of TMA. Subsequently, TMA is oxidized in the liver by the enzyme FMO3, resulting in the formation of TMAO.^24^ Elevated levels of TMAO have been linked to a heightened risk of cognitive impairments,^25^, ^26^ which was also observed in our present study. It's possible that TMAO may be explored as a diagnostic marker for POCD post‐hip replacement surgery but this point should be confirmed by a large clinical cohort in the future. In our investigation, a consistent elevation of TMAO levels was observed across different samples from mice with POCD, with intestinal TMAO concentrations positively associating with the extent of cognitive impairment. The consistency in the alterations of TMAO levels in the gut, plasma, and brain tissues indicates the possibility that increased TMAO in the gut may result in the harmful exposure of central nervous system tissues to microbial products via the bloodstream (gut‐brain axis). This exposure could lead to an escalated inflammatory response in the brain, consequently impairing neural functions.^27^ The intrinsic connection between TMAO levels and cognitive function in postoperative mice is further supported by the observable cognitive changes induced by variations in TMAO levels. Specifically, the reduction in TMAO levels following pretreatment with ABX coincides with the mitigation of cognitive impairments in postoperative mice. Conversely, we utilized FMT from donors with the femoral fracture operation model as a technique to modify the diversity and metabolic functions of the gut microbiome in recipients. This intervention modulated the abundance of TMAO in the recipients, resulting in noticeable changes in cognitive function. These findings, from both positive and negative perspectives, highlight the significant impact of TMAO on cognitive functions post‐surgery, illustrating the direct interplay between TMAO and the neurocognitive outcome. It is also noteworthy that this process shows a certain degree of dependency on pro‐inflammatory factors in brain tissue, indicating a close relationship between the effects of TMAO and neuroinflammation, which is identified as one of the main pathologic processes of POCD.^28^

In the further exploration of the mechanisms through which TMAO modulates POCD, our investigation revealed mice directly treated with TMAO showed not only a pronounced increase in the characteristics of POCD but also experienced activation and polarization changes in microglia. After being exposed to external stimuli, such as surgical injury or harmful substances like certain intestinal metabolites, the microglia can promote inflammatory responses that inflict damage on neurons and consequently impair cognitive functions.^29^ To determine whether microglia play an indispensable role in the neurotoxic effects of TMAO, the microglial ablation agent was utilized to provide further evidence.^30^ PLX5622‐treated mice depicted a significant decrease in cognitive deficits and neuroinflammatory cytokines, highlighting the potential therapeutic value of targeting microglial activation to reduce the damage to the nervous system induced by TMAO. The regulatory mechanism of certain gut metabolites and their metabolites on cognitive functions through microglia has been extensively reported in previous research.^31^ For instance, *Clostridium butyricum* can alleviate microglia‐mediated neuroinflammation by regulating the GM‐gut‐brain axis mediated through the metabolite butyrate.^32^ However, our present study is the first to report on its regulatory effect on the exacerbation of POCD induced by TMAO. Several studies showed that TMAO crosses the blood–brain barrier easily reaching the brain.^33^, ^34^ As a result, we speculated that TMAO may directly interact with microglia. Remarkably, a recent study showed that TMAO treatment could induce microglial activation toward a proinflammatory phenotype.^35^ These studies may offer insights into the potential mechanisms by which TMAO activates microglia, but the specific mechanism still requires further exploration in future research.

While this study provides substantial evidence of TMAO's involvement in exacerbating POCD through the modulation of microglial activation, several limitations need to be considered. As mice do not perfectly replicate human physiological responses or cognitive processes. The translational relevance of these findings to clinical settings necessitates cautious interpretation. Furthermore, the direct measurement of cognitive function in mice through behavioral tests, although informative, may not fully capture the breadth of cognitive impairments experienced by patients with POCD. Further research is required to address these limitations, including studies that utilize diverse animal models and perform more comprehensive assessments of cognitive functions. Moreover, analyzing the correlation between serum and intestinal TMAO levels with the incidence of POCD in elderly patients undergoing hip replacement surgery would strengthen our current findings. This will provide scientific information for developing TMAO‐targeted treatment strategies for patients with POCD post‐hip replacement surgery.

In conclusion, our research elucidates the significant role of TMAO in the exacerbation of POCD following femoral fracture surgery in mice and shows the interconnection among gut microbiota, TMAO, and the consequent cognitive impairments. Furthermore, direct TMAO treatment could worsen cognitive deficits and neuroinflammation in the context of POCD, where microglial activation plays a crucial role. Our study provides valuable insights into the mechanisms by which TMAO contributes to cognitive dysfunction in postoperative mice, identifying potential targets for therapeutic intervention and strategies to enhance the quality of life for elderly patients.

## CONFLICT OF INTEREST STATEMENT

The authors declare no conflict of interest.

## Supporting information


Figure S1
