# Supplementary material for: Gut microbiota‐derived metabolite trimethylamine N‐oxide aggravates cognitive dysfunction induced by femoral fracture operation in mice
Source: Kaohsiung J Med Sci. 2024 Jul 4;40(8):732–43. doi: 10.1002/kjm2.12873 (PMC11895615; doi:10.1002/kjm2.12873)
Supplement: Supplementary file 1 — Figure S1 [file KJM2-40-732-s001.zip › FigureS1_Legend.docx]

Figure S1 Effects of TMAO treatment on the liver structure and TMAO levels in a femoral fracture operation mouse model. (A) H&E staining of liver sections. (B) The TMAO levels in plasma, brain, and intestines (left to right).
