# Supplementary figures and images for: Gut microbiota‐derived metabolite trimethylamine N‐oxide aggravates cognitive dysfunction induced by femoral fracture operation in mice
Source: Kaohsiung J Med Sci. 2024 Jul 4;40(8):732–43. doi: 10.1002/kjm2.12873 (PMC11895615; doi:10.1002/kjm2.12873)

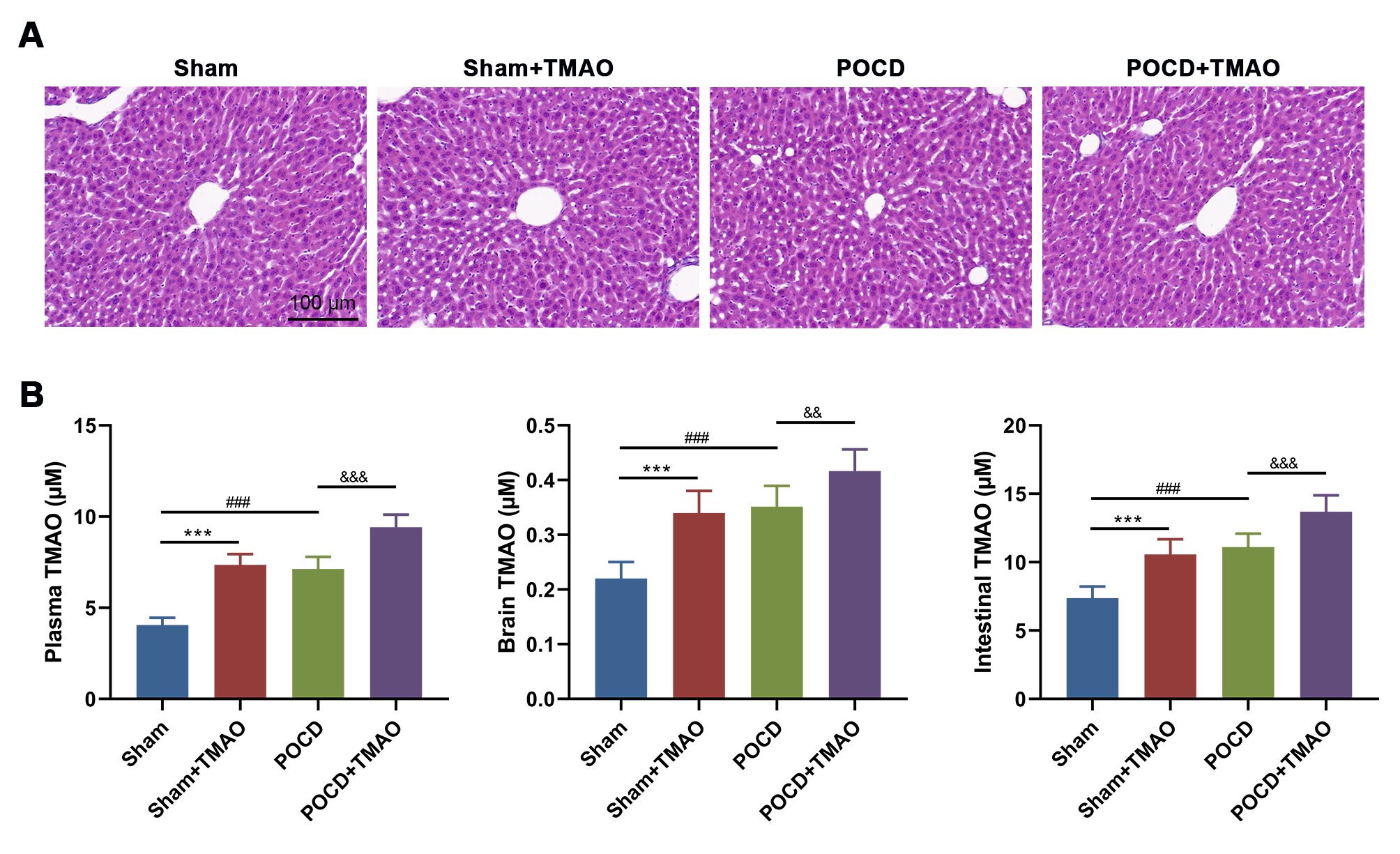

Supplement: Supplementary file 1 — Figure S1 [file KJM2-40-732-s001.zip › kjm212873-sup-0001-FigureS1.tif]
